# Supplementary material for: Characterization of 22q12 Microdeletions Causing Position Effect in Rare NF2 Patients with Complex Phenotypes
Source: Int J Mol Sci. 2022 Sep 2;23(17):10017. doi: 10.3390/ijms231710017 (PMC9456353; doi:10.3390/ijms231710017)
Supplement: Supplementary file 1 [file ijms-23-10017-s001.zip › Table S1.pdf]

Table S1. Deletion gene content

Patient	246	366	160
**Proximal BP**	chr22:29,928,889-29,928,896	chr22:29,594,735-29,594,767	chr22:29,277,234-29,277,248
**Distal BP**	chr22:30,075,777-30,075,784	chr22:30,155,876-30,155,908	chr22:31,143,939-31,143,953
**Gene content**	*THOC5, NIPSNAP1, NF2*	*EMID1, RHBDD3, EWSR1, GAS2L1, RASL10A, AP1B1, RFPL1, NEFH, THOC5, NIPSNAP1, NF2, CABP7, ZMAT5*	*ZNRF3, C22orf31, KREMEN1, EMID1, RHBDD3, EWSR1, GAS2L1, RASL10A, AP1B1, RFPL1, NEFH, THOC5, NIPSNAP1, NF2, CABP7, ZMAT5, UQCR10, ASCC2, MTMR3, HORMAD2, LIF, OSM, CASTOR1, TBC1D10A, SF3A1, CCDC157, RNF215, SEC14L2, MTFP1, SEC14L3, SEC14L4, SEC14L6, GAL3ST1, PES1, TCN2, SLC35E4, DUSP18, OSBP2*
