# Supplementary material for: Characterization of 22q12 Microdeletions Causing Position Effect in Rare NF2 Patients with Complex Phenotypes
Source: Int J Mol Sci. 2022 Sep 2;23(17):10017. doi: 10.3390/ijms231710017 (PMC9456353; doi:10.3390/ijms231710017)
Supplement: Supplementary file 1 [file ijms-23-10017-s001.zip › Table S2.pdf]

Table S2. Statistical analysis of real-time PCR data

Gene	Samples	2^(−ΔCt) mean ± SD	BH-adjusted p > ItI	
			PT *vs* WT	PT *vs* NF2im
AP1B1	WT	0.214 ± 0.024		
	NF2im	0.218 ± 0.110		
	PT 246	0.131 ± 0.072	0.034	0.570
MTMR3	WT	1.076 ± 0.211		
	NF2im	1.074 ± 0.563		
	PT 246	2.190 ± 1.052	0.036	0.464
	PT 366	1.136 ± 0.698	0.974	0.965
LIMK2	WT	0.454 ± 0.241		
	NF2im	0.303 ± 0.022		
	PT 246	0.789 ± 0.139	0.137	0.024
	PT 366	0.393 ± 0.125	0.954	0.568
	PT 160	0.504 ± 0.014	0.942	0.003
PIK3IP1	WT	0.295 ± 0.127		
	NF2im	0.268 ± 0.028		
	PT 246	0.235 ± 0.074	0.746	0.762
	PT 366	0.287 ± 0.152	0.930	0.942
	PT 160	0.399 ± 0.021	0.444	0.026

NF2im, patients with *NF2* intragenic mutation; BH-adjusted p, p-value corrected by Benjamini-Hochberg method; PT, patient; SD, standard deviation; t, t-value; WT, wild type controls
