# Supplementary material for: Characterization of 22q12 Microdeletions Causing Position Effect in Rare NF2 Patients with Complex Phenotypes
Source: Int J Mol Sci. 2022 Sep 2;23(17):10017. doi: 10.3390/ijms231710017 (PMC9456353; doi:10.3390/ijms231710017)
Supplement: Supplementary file 1 [file ijms-23-10017-s001.zip › Table S3.pdf]

**Table S3. Regulatory elements mapping in 22q12 region**

| Category | ID | Genomic coordinates | PT 246 deletion | PT 366 deletion | PT 160 deletion |
|---|---|---|---|---|---|
| Enhancer | hs1645 | chr22:28,746,697-28,748,395 | out | out | out |
| Enhancer | hs2240 | chr22:32,019,365-32,023,822 | out | out | out |
| Insulator | EMBL_M1 | chr22:30,682,932-30,682,945 | out | out | in |
| Insulator | EMBL_M1 | chr22:30,783,441-30,783,454 | out | out | in |
| Insulator | EMBL_M2 | chr22:28,359,682-28,359,690 | out | out | out |
| Insulator | EMBL_M2 | chr22:28,469,178-28,469,186 | out | out | out |
| Insulator | EMBL_M2 | chr22:28,543,844-28,543,852 | out | out | out |
| Insulator | EMBL_M2 | chr22:28,698,163-28,698,171 | out | out | out |
| Insulator | EMBL_M2 | chr22:28,758,324-28,758,332 | out | out | out |
| Insulator | EMBL_M2 | chr22:28,760,291-28760299 | out | out | out |
| Insulator | EMBL_M2 | chr22:28,847,448-28,847,456 | out | out | out |
| Insulator | EMBL_M2 | chr22:28,864,987-28,864,995 | out | out | out |
| Insulator | EMBL_M2 | chr22:29,041,938-29,041,946 | out | out | out |
| Insulator | EMBL_M2 | chr22:29,090,446-29,090,454 | out | out | out |
| Insulator | EMBL_M2 | chr22:29,210,918-29,210,926 | out | out | out |
| Insulator | EMBL_M2 | chr22:29,298,811-29,298,819 | out | out | in |
| Insulator | EMBL_M2 | chr22:29,788,637-29,788,645 | out | in | in |
| Insulator | EMBL_M2 | chr22:30,083,878-30,083,886 | out | in | in |
| Insulator | EMBL_M2 | chr22:30,093,309-30,093,317 | out | in | in |
| Insulator | EMBL_M2 | chr22:30,206,272-30,206,280 | out | out | in |
| Insulator | EMBL_M2 | chr22:30,334,088-30,334,096 | out | out | in |
| Insulator | EMBL_M2 | chr22:30,697,806-30,697,814 | out | out | in |
| Insulator | EMBL_M2 | chr22:30,766,430-30,766,438 | out | out | in |
| Insulator | EMBL_M2 | chr22:30,833,966-30,833,974 | out | out | in |
| Insulator | EMBL_M2 | chr22:30,928,154-30,928,162 | out | out | in |
| Insulator | EMBL_M2 | chr22:30,986,847-30,986,855 | out | out | in |
| Insulator | EMBL_M2 | chr22:31,041,156-31,041,164 | out | out | in |
| Insulator | EMBL_M2 | chr22:31,047,945-31,047,953 | out | out | in |
| Insulator | EMBL_M2 | chr22:31,336,929-31,336,937 | out | out | out |
| Insulator | EMBL_M2 | chr22:31,401,652-31,401,660 | out | out | out |
| Insulator | EMBL_M2 | chr22:31,483,953-31,483,961 | out | out | out |
| Insulator | EMBL_M2 | chr22:31,548,528-31,548,536 | out | out | out |
| Insulator | EMBL_M2 | chr22:31,663,502-31,663,510 | out | out | out |
| Insulator | REN_20 | chr22:30,783,438-30,783,457 | out | out | in |
| Insulator | MIT_LM2 | chr22:30,881,492-30,881,510 | out | out | in |
| Insulator | MIT_LM7 | chr22:29,808,882-29,808,901 | out | in | in |
| Insulator | MIT_LM23 | chr22:31,478,757-31,478,776 | out | out | out |
