# Supplementary material for: Characterization of 22q12 Microdeletions Causing Position Effect in Rare NF2 Patients with Complex Phenotypes
Source: Int J Mol Sci. 2022 Sep 2;23(17):10017. doi: 10.3390/ijms231710017 (PMC9456353; doi:10.3390/ijms231710017)
Supplement: Supplementary file 1 [file ijms-23-10017-s001.zip › Table S4.pdf]

Table S4. Sequence of primers for qPCR-gDNA assays

Region target	Oligonucleotide name	Sequence (5′ to 3′)
chr22:25,767,231-25,767,386	LRP5L_gDNA F	AAGGCAAGAAGGAACAGCAC
	LRP5L_gDNA R	TCCAGAGAGCCCCTTACTC
chr22:27,006,914- 27,007,085	CRYBB1_gDNA F	TGCCTGTTCCTGTCTCTGTG
	CRYBB1_gDNA R	GGGAGACAAGAGCAAACGAG
chr:28,190,925-28,191,140	MN1_gDNA F	CAGCATCAGCATTCTCCAGG
	MN1_gDNA R	TAGAAGTGACAGGGGCTGAG
chrr22:29,386,019-29,386,193	ZNRF3_gDNA F	CATTCTGTGGGAGGAGGTAC
	ZNRF3_gDNA R	GCCTCTCCACATTAAGTACAG
chr22:29,498,291-29,498,462	KREMEN1_gDNA F	GAGTGTGCCTTCATCCAGTC
	KREMEN1_gDNA R	AATGGACCGAGGAAAATCTGC
chr22:29,727,322-29,727,479	AP1B1_gDNA F	CGACTCCATTTACGGGCAAG
	AP1B1_gDNA R	TGTACCCACTGCACATCCTC
chr22:29,840,970-29,841,120	RFPL1_NEFH_gDNA F	GCCCATACCCTTAGCTGTCA
	RFPL1_NEFH_gDNA R	CACCTTCTTCTGCACGACCA
ch22:30,051,058-30,051,176	NF2 intron 5_gDNA F	AGAGGAGATGAGGTAGGGGT
	NF2 intron 5_gDNA R	GAGGGCTGTAAAATCGGCAG
chr12:6,643,799-6,643,932	GAPDH_gDNA F	CAGGCCGGATGTGTTCGC
	GAPDH_gDNA R	GACACACACGCCTCCCCT
