# Supplementary material for: Characterization of 22q12 Microdeletions Causing Position Effect in Rare NF2 Patients with Complex Phenotypes
Source: Int J Mol Sci. 2022 Sep 2;23(17):10017. doi: 10.3390/ijms231710017 (PMC9456353; doi:10.3390/ijms231710017)
Supplement: Supplementary file 1 [file ijms-23-10017-s001.zip › Table S5.pdf]

Table S5. Sequence of primers for Long-range PCR assays

Repeated sequence	Oligonucleotide name	Sequence (5′ to 3′)	Paatient tested
MLT1K	MLT1K F	CTGCAGCAAACTATGATGAAG	246
MIRb	MIRb F	AGTCTGGGTGAAAGGGTAAG	246
L1ME3	THOC5 int 7 F	TAACCCAAAAGAGGCCCATG	246
	THOC5 int 8 F	TGACGGACACAGAGAACCC	246
	THOC5 int 8 (N) F	CCTGTAATCCCAGCACTTTG	246
	THOC5 int 9 F	ACAGTAGCTGCCCCATTAGT	246
	THOC5 int 9 (N) F	TGGCTCATGTCTGGTTGGGA	246
MLT1A0	MLT1A0 a R	TGGTCTCAAAGGGCTGGGA	246
	MLT1A0 b R	TCTCCTTGTCTCAACCCTTC	246
	MLT1A0 c R	ACCCCTCATCCCTTCCACT	246
	MLT1A0 c (N) R	TAGACTCCACCTCTGCCCG	246
	MLT1A0 c (N2) R	TGAGACAGGGTGTCTCTCTG	246
L2a	L2a F	TGTGCCTGGCCTATGTTGAC	366
L1MC4	L1MC4a F	GGTCCCTCAGTTCACCTTTC	366
	L1MC4a (N1) F	CCTAGTGTGGGTTTCTCTTAT	366
	L1MC4a (N2) F	AGTAGTCTCACTCTGTCACTC	366
Tigger2b_Pri	Tigger2b F	ACAATATGGGGATTAAGGCAC	366
MIRb	MIRb(1) F	GAGAGGAGCCACGATTCAC	366
AluJb	AluJb(2) R	TGGATTCAGACCTGGGTTGG	366
MLT1F	MLT1F R	CACTGAGATGGAAGCAGCAG	366
AluSx	AluSx(2) R	CAGGGAGTTAAAGGTGTGGC	366
AluJb	AluJb(1) R	CGAGATCACCTAGTCCCAG	366
MIRb	MIRb R	AGTGCCAAGCAAGCGGGAA	366
AluSx	AluSx(1) R	TCCAGGAGTGTGACAAGACA	366
	AluSx(1) (N1) R	AAGCGATTCTCCTGCCTCAG	366
	AluSx(1) (N2) R	GAATCTCGCTCTGTTGCCC	366
L2b	L2b R	GCTCAACTCTTCGCCTTGTC	366
AluJb	AluJb F	TTGTTCCCACCACATGACTTA	160
MLT1K	MLT1K F	CAAGCCTTGAATGCCAGGAT	160
MER102c	MER102c F	TCCATCATCAGCCTTTCTTGG	160
MER2b	MER2b F	TTTCAGATGTAAGCCACCGC	160
L1MB7	L1MB7 F	GCAGGCCAAGGGAGGATAG	160
	L1MB7 (N) F	TTGAGGGTGTGAAATGTGCG	160
	L1MB7 (N2) F	GGGCTCAGGAGTTTGATTCC	160
	L1MB7 (N3)F	AGAATATGTGGGCTTTTGGGT	160
	L1MB7 (N4) F	TGGAGTCTCACTCTGTCGC	160
AluSx	AluSx F	TCCAGGTTCTTGCATGTTGT	160
MLT1C	MLT1C F	GCTGGGCTCTCTATCCTTC	160
L1ME34	L1ME34 R	ATAGGCACCCACCAATATGC	160

AluJb	AluJb R	TCAACAGAGTGAGATGAGACT	160
AluSz	AluSz R	CTGCTCAAACTTGGGAGAAG	160
LTR16D2	LTR16D2 R	GAGGGTCTAGGGTGGGTAG	160
L2b	L2b R	TGCTCCTTTTGCTTTCTGAGG	160
	L2b (N) R	CGCAACACCACAGTCTTCTT	160
