# Supplementary material for: Characterization of 22q12 Microdeletions Causing Position Effect in Rare NF2 Patients with Complex Phenotypes
Source: Int J Mol Sci. 2022 Sep 2;23(17):10017. doi: 10.3390/ijms231710017 (PMC9456353; doi:10.3390/ijms231710017)
Supplement: Supplementary file 1 [file ijms-23-10017-s001.zip › Table S6.pdf]

Table S6. Sequence of primers for PCR assays

Oligonucleotide name	Sequence (5′ to 3′)
AP1B1_1_FW	CGGACATTTTGGTGCCAAGC
AP1B1_1_REV	CTCACAGTCCTTCACAAAGGT
AP1B1_2_FW	AGATGTCAGTGCCCTCTTCC
AP1B1_2_REV	GGGTGAGACTCGGCAATTTC
AP1B1_3_FW	CCAAGCTCCACGACATCAAC
AP1B1_3_REV	TCTCAGGCCTTTTCTGCACG
AP1B1_4_FW	CTGCTGTGAAGGTGCTGATG
AP1B1_4_REV	ACACTCTCATACTTGTTGGGG
AP1B1_5_FW	CGCAACAGAAGTGGATGTGG
AP1B1_5_REV	TATCTGAGTCCTGAGTGGCC
AP1B1_6.1_FW	GCTTCCATGACGAGAGCACA
AP1B1_6.2_FW	CCCACACTGTTAGACGAGCT
AP1B1_6.1_REV	CATCCCCCATCAGGCTGTC
AP1B1_6.2_REV	GTTGGTGCCCCCAATCAGG
AP1B1_7.1_FW	CAGCCTGATGGGGGATGAG
AP1B1_7.2_FW	CCTGATTGGGGGCACCAAC
AP1B1_7_REV	AGGGAGATCTCCACTGTCTG
AP1B1_8_FW	CCCCAAAGCAGTCTGGCTCC
AP1B1_8_REV	GATGTTGCTGCTCTGCAGCT
AP1B1_9_FW	ACGGGAAGATGGACCGGCA
AP1B1_9.1_REV	CTAAGTCCGTGCAGCTGGG
AP1B1_9.2_REV	TCAGGGACAGCGTGCAGCT
AP1B1_10.1_FW	CCCAGCTGCACGGACTTAG
AP1B1_10.2_FW	AGCTGCACGCTGTCCCTGA
AP1B1_10_REV	TAACCCCACATCACAGCCAC
