# Supplementary material for: Characterization of 22q12 Microdeletions Causing Position Effect in Rare NF2 Patients with Complex Phenotypes
Source: Int J Mol Sci. 2022 Sep 2;23(17):10017. doi: 10.3390/ijms231710017 (PMC9456353; doi:10.3390/ijms231710017)
Supplement: Supplementary file 1 [file ijms-23-10017-s001.zip › Table S7.pdf]

Table S7. Sequence of primers for RT-qPCR assays

Gene target	Oligonucleotide name	Sequence (5′ to 3′)
HSCB	HSCB_qPCR F	GCGTCTTGTCCACCCAGATT
	HSCB_qPCR R	GGAGCTTTAGAAGGTACAGTC
CCDC117	CCDC117_qPCR F	AGAAACACAAGCGAGAGGAG
	CCDC117_qPCR R	TTACTCCATGCCCCTCTACA
XBP1	XBP1_qPCR F	GCCAAGGGGAATGAAGTGAG
	XBP1_qPCR R	ATGGGGAGATGTTCTGGAGG
AP1B1	AP1B1_qPCR F	CCTGGTCACACTGCTGTCA
	AP1B1_qPCR R	TCAGGCCTTTTCTGCACGAT
ZMAT5	ZMT5_qPCR F	ACAACCTCCACAACCGCAAG
	ZMT5_qPCR R	CTGCATCTCGGAACATGTCG
UQCR10	UQCR10_qPCR F	TTGTACTCCCTGCTGTTCCG
	UQCR10_qPCR R	CTTCCCCTCGTTGATGTGGT
ASCC2	ASCC2_qPCR F	ACCCGAAGACAGGAAAGCTG
	ASCC2_qPCR R	CACTAGGGCGGGAATGTTGT
MTMR3	MTMR3_qPCR F	GTGGCTTGACTGTGTTCATC
	MTMR3_qPCR R	TGCTTTTCCCCTCTCTCCTT
RNF185	RNF185_qPCR F	GCAGGACAGCACTTTCGAG
	RNF185_qPCR R	ACCACTGATGTAAACACGGC
LIMK2	LIMK2_qPCR F	CTCTTGCTTCCGGTGTTCAG
	LIMK2_qPCR R	GCAGTAGAGCTTCCCATCC
PIK3IP1	PIK3IP1_qPCR F	GGGTACAAGCATTCCTCGTC
	PIK3IP1_qPCR R	GTTGTCCCAGAAACAGCCTC
PATZ1	PATZ1_qPCR F	GCCTGATCACTTGAACGGAC
	PATZ1_qPCR R	GTCTCGGGTGGCAAAAGAAG
EIF4A2	EIF4A2_qPCR F	GGTCAGGGTCAAGTCGTGTT
	EIF4A2_qPCR R	CCCCCTCTGCCAATTCTGTG
