# Supplementary figures and images for: Characterization of 22q12 Microdeletions Causing Position Effect in Rare NF2 Patients with Complex Phenotypes
Source: Int J Mol Sci. 2022 Sep 2;23(17):10017. doi: 10.3390/ijms231710017 (PMC9456353; doi:10.3390/ijms231710017)

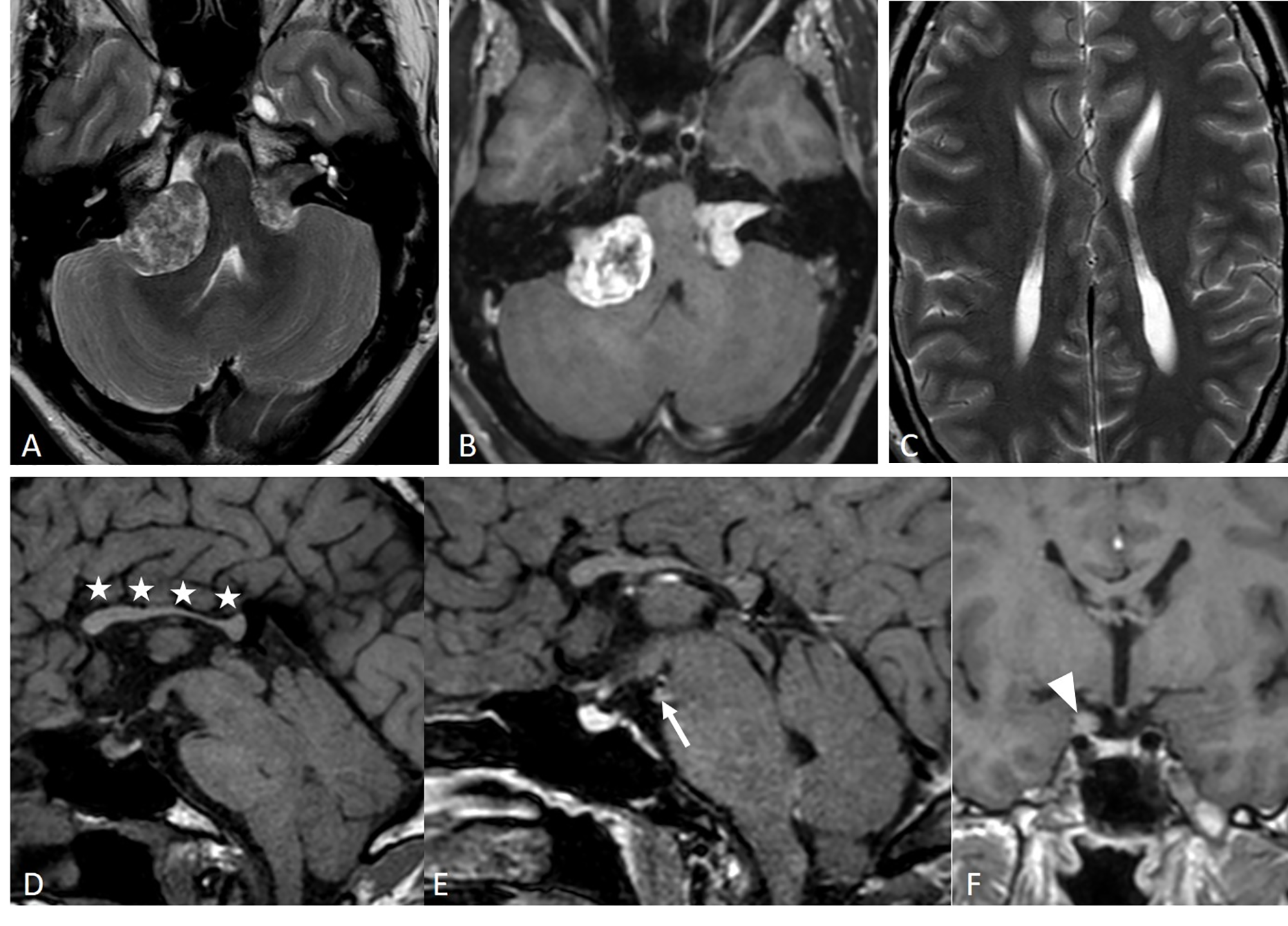

Supplement: Supplementary file 1 [file ijms-23-10017-s001.zip › Figure S1.tif]

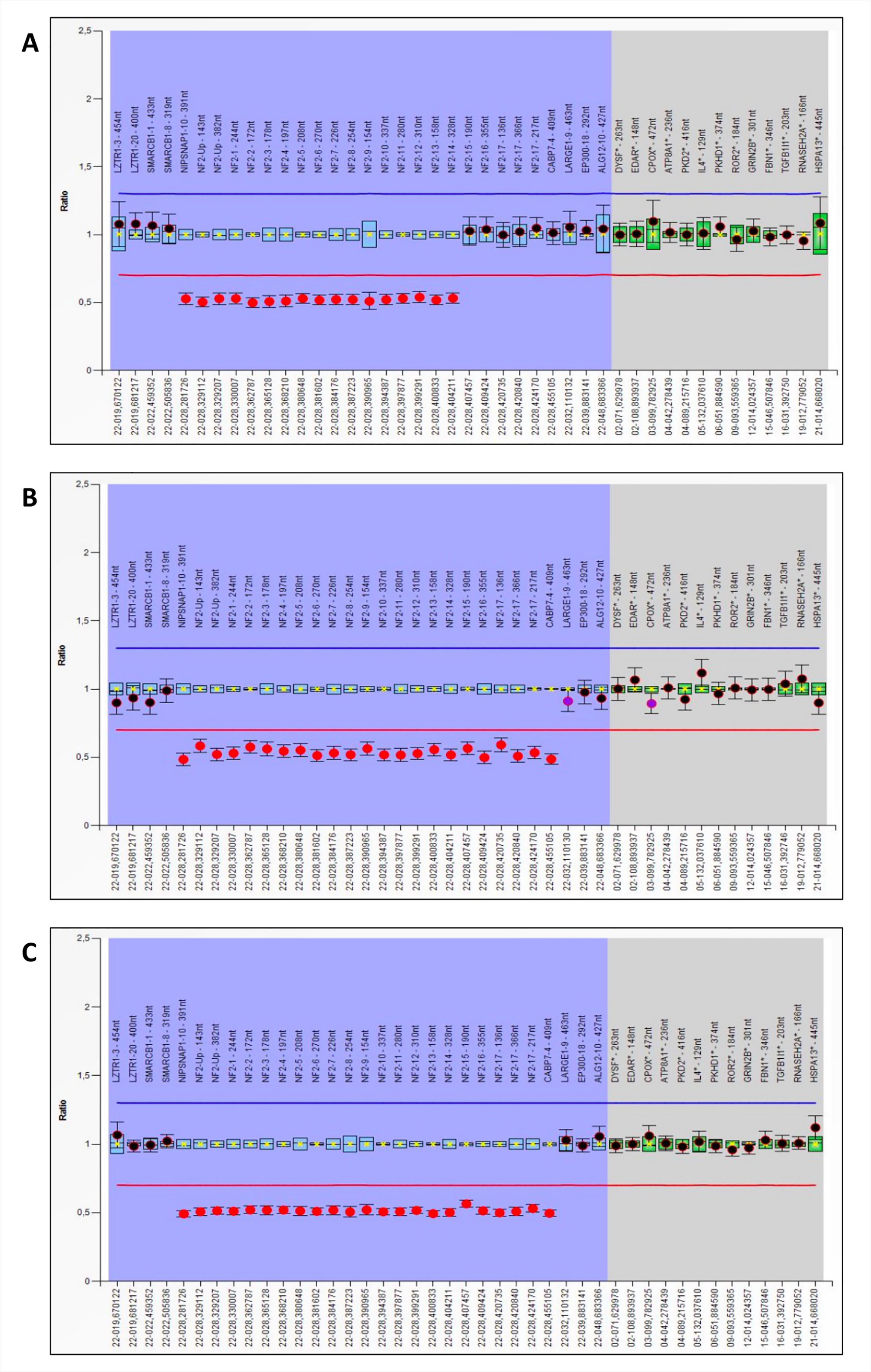

Supplement: Supplementary file 1 [file ijms-23-10017-s001.zip › Figure S2.tif]

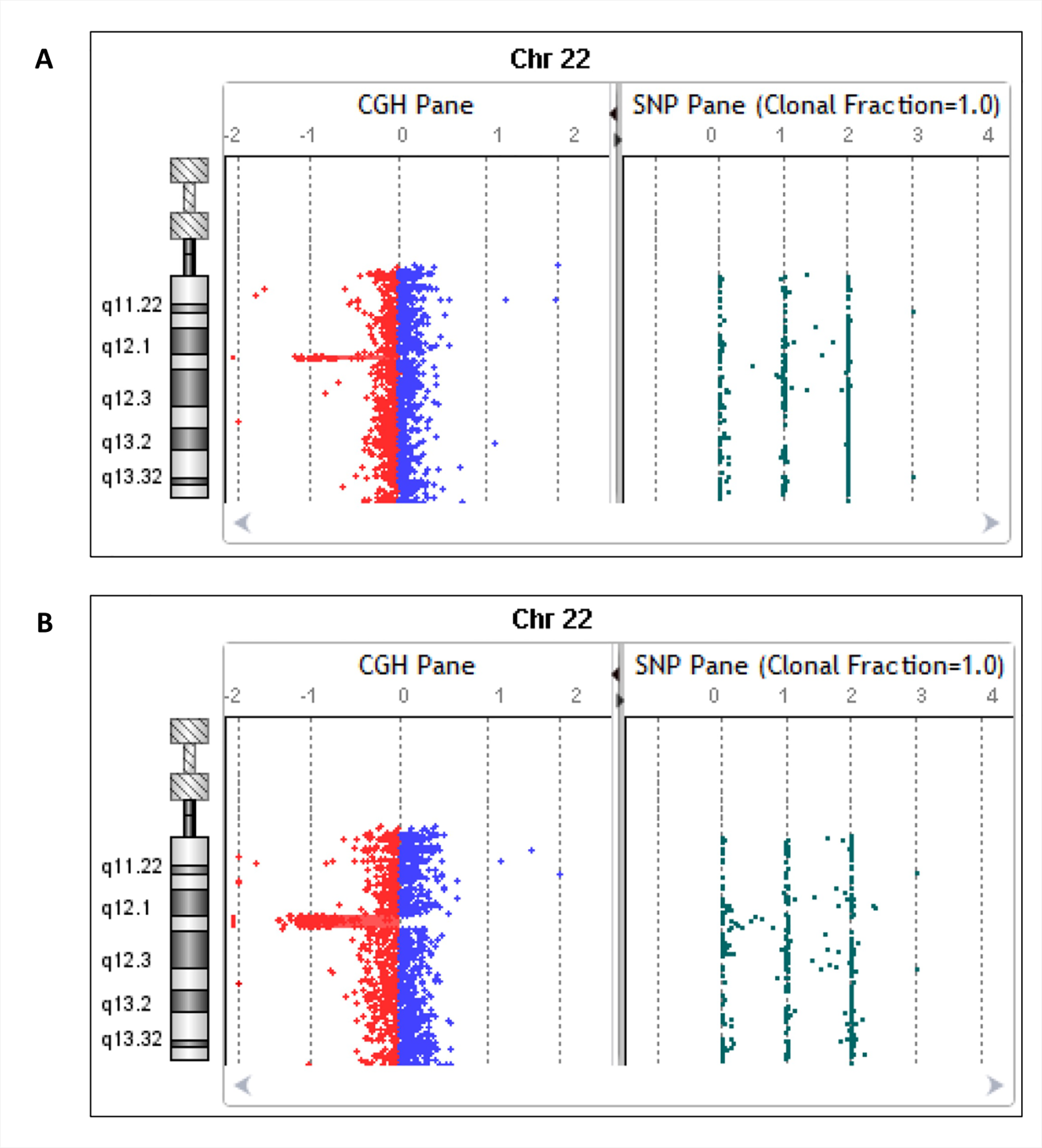

Supplement: Supplementary file 1 [file ijms-23-10017-s001.zip › Figure S4.tif]

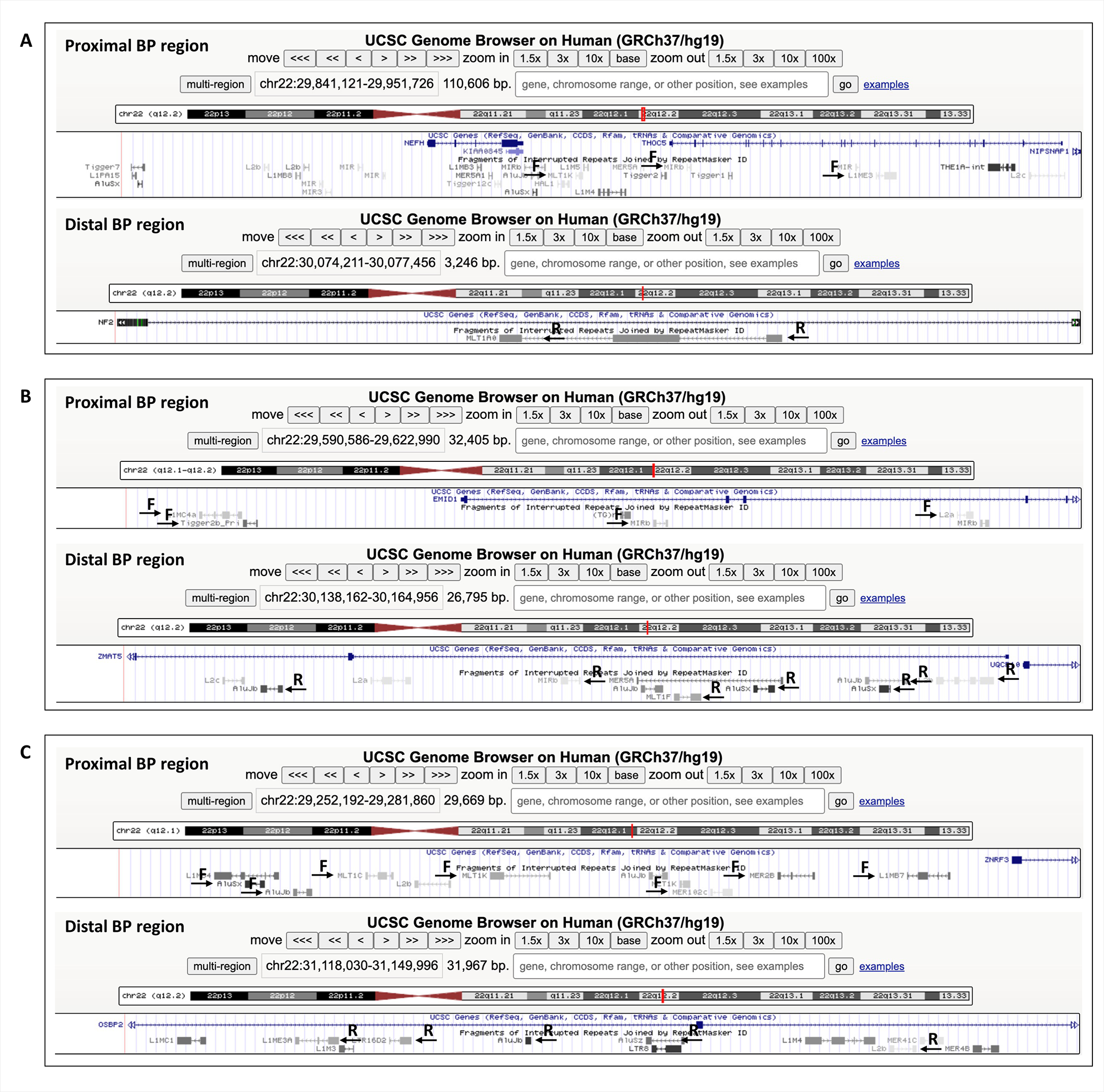

Supplement: Supplementary file 1 [file ijms-23-10017-s001.zip › Figure S5.tif]
